# Overview of Efforts to Increase Women Enrollment in the Veterans Affairs Million Veteran Program

**DOI:** 10.1089/heq.2023.0006

**Published:** 2023-05-26

**Authors:** Stacey B. Whitbourne, Yanping Li, Jessica V.V. Brewer, Jennifer Deen, Claudia Gutierrez, Sybil A. Murphy, Emily Lord, Joseph Yan, Xuan-Mai T. Nguyen, Philip S. Tsao, J. Michael Gaziano, Sumitra Muralidhar

**Affiliations:** ^1^ Million Veteran Program Coordinating Center, VA Boston Healthcare System, Boston, Massachusetts, USA.; ^2^ Department of Medicine, Harvard Medical School, Boston, Massachusetts, USA.; ^3^ Division of Aging, Department of Medicine, Brigham and Women's Hospital, Boston, Massachusetts, USA.; ^4^ Office of Research and Development, Veterans Health Administration, Washington, District of Columbia, USA.; ^5^ Million Veteran Program Coordinating Center, VA Palo Alto Health Care System, Palo Alto, California, USA.; ^6^ Carle Illinois College of Medicine, University of Illinois, Champaign, Illinois, USA.; ^7^ Department of Medicine, Stanford University School of Medicine, Stanford, California, USA.; ^8^ Stanford Cardiovascular Institute, Stanford University School of Medicine, Stanford, California, USA.

**Keywords:** Veterans, Million Veteran Program, women, recruitment

## Abstract

**Background:**

Ensuring enhanced delivery of care to women Veterans is a top Veterans Affairs (VA) priority; however, women are historically underrepresented in research that informs evidence-based health care. A primary barrier to women's participation is the inability to engage with research in person due to a number of documented challenges. The VA Million Veteran Program (MVP) is committed to increasing access for women Veterans to participate in research, thereby better understanding conditions specific to this population and how disease manifests differently in women compared to men. The goal of this work is to describe the results of the MVP Women's Campaign, an effort designed to increase outreach to and awareness of remote enrollment options for women Veterans.

**Materials and Methods:**

The MVP Women's Campaign launched two phases between March 2021 and April 2022: the Multimedia Phase leveraged a variety of strategic multichannel communication tactics and the Email Phase focused on direct email communication to women Veterans. The effect of the Multimedia Phase was determined using *t*-tests and chi-square tests, as well as logistic regression models to compare demographic subgroups. The Email Phase was evaluated using comparisons of the enrollment rate across demographic groups through a multivariate adjusted logistic regression model.

**Results:**

Overall, 4694 women Veterans enrolled during the MVP Women's Campaign (54% during the Multimedia Phase and 46% during the Email Phase). For the Multimedia Phase, the percentage of older women online enrollees increased, along with women from the southwest and western regions of the United States. Differences for women Veteran online enrollment across different ethnicity and race groups were not observed. During the Email Phase, the enrollment rate increased with age. Compared to White women Veterans, Blacks, Asians, and Native Americans were significantly less likely to enroll while Veterans with multiple races were more likely to enroll.

**Conclusion:**

The MVP Women's Campaign is the first large-scale outreach effort focusing on recruitment of women Veterans into MVP. The combination of print and digital outreach tactics and direct email recruitment resulted in over a fivefold increase in women Veteran enrollees during a 7-month period. Attention to messaging and communication channels, combined with a better understanding of effective recruitment methods for certain Veteran populations, allows MVP the opportunity to advance health and health care not only for women Veterans, but beyond. Lessons learned will be applied to increase other populations in MVP such as Blacks, Hispanics, Asians, Native Americans, younger Veterans, and Veterans with certain health conditions.

## Introduction

Women Veterans make up the fastest growing segment of Veterans Health Administration (VHA) users. From 2005 to 2015, women Veterans accessing VHA care nearly tripled.^1^ As such, ensuring delivery of comprehensive, evidence-based women's care is a key VHA priority, given that its health care system predominantly delivers services to men, who comprise over 90% of the Veteran population. Research focusing on enhanced treatment for women Veterans has also increased in recent years^2^; an important step in helping to overcome the historical lack of women represented in research overall. This imbalance limits understanding gender-based differences in Veterans and their impact on health, especially as women Veterans vary both from a demographic and clinical care perspective.

Women Veterans are younger, more racially and ethnically diverse, and are 20% more likely to utilize mental health services compared to men.^3^ Access to care for women Veterans, particularly for mental health services, is also faced with challenges, ranging from lack of family care and travel issues to reports of harassment experienced in waiting rooms.^4,5^ Offering remote methods for women to receive care (e.g., through telehealth or virtual options) has garnered support in an effort to remove barriers reported by women Veterans.^2^ Likewise, remote participation opportunities should also be offered to engage more women Veterans in research.

The Department of Veterans Affairs (VA) Million Veteran Program (MVP), launched in 2011, is one of the world's largest and most comprehensive cohorts of genetic, health record, lifestyle, and military experience information with a focus on understanding how these factors impact the health and wellness of Veterans.^6,7^ As of October 2022, over 912,000 Veterans are enrolled in MVP, with participant demographics closely matching VHA users. To date, ∼90,000 women Veterans enrolled in MVP, representing close to 10% of the overall cohort. One of MVP's priorities is to over-index women in the cohort, thereby increasing statistical power needed for meaningful genetic research on conditions important to this population.

In 2019, MVP launched an online platform enabling at-home enrollment, including informed consent, survey completion, and blood specimen collection arrangements. While ∼97% of MVP enrollments continue to occur at VA facilities, the online option for enrollment has increased access for many Veterans, including underrepresented populations in research. As of November 2021, Veterans who enroll online can have blood collection devices mailed directly to them, allowing for full remote enrollment.

MVP offers a unique opportunity to better understand conditions, particularly genetic conditions, in areas specific to women Veterans, such as breast cancer and mental health, which affect more women than men in the Veteran community. Previous work has described gender differences in the demographic and health characteristics of MVP, with small-scale pilot work on focused recruitment of women Veterans.^8^

In 2021, MVP launched a recruitment campaign to increase enrollment of women Veterans focusing on the ability to enroll online, to support expanding research into women Veterans' health. The goal of this work is to describe the results of the MVP Women's Campaign overall and stratified by demographic characteristics.

## Materials and Methods

The VA Central Institutional Review Board is responsible for oversight and approval of MVP (no. 10-02). The MVP Women's Campaign comprised two phases which took place between March 2021 and April 2022. The Multimedia Phase focused on leveraging a variety of multichannel communication strategies such as social and traditional media, mass email, blogs, videos, podcasts, newsletters, print outreach materials, and presentations to partners and stakeholders. The Email Phase focused on direct email communication to women Veterans. Key messages for all campaign materials focused on highlighting the importance and potential impacts of increasing the representation and inclusion of women Veterans in health research, including how participation in MVP may help advance breakthroughs in women Veteran's health care.

### Multimedia phase

The Multimedia Phase ran from March 2021 to August 2021 and involved ∼90 activities ranging from a national VA press release; an outreach toolkit with materials such as posters, social media posts, digital billboards distributed to all 171 VA medical centers and women Veteran-focused Veteran Service Organizations (VSOs); podcast episodes; a Facebook Live event; local feature stories; public service announcements; a VA News episode; and an email campaign distributed by the VA Veteran Experience Office.

To evaluate the effect of the Multimedia Phase on enrollment of women Veterans online, the crude difference of MVP enrollment online was compared before and after the Multimedia Phase across different demographic groups using *t*-tests for continuous variables and chi-square tests for categorical characteristics. A multivariate adjusted comparison with mutual adjustment of age, ethnicity, race, geographic area, and service era was conducted using logistic regression models comparing each demographic subgroup with the reference group.

### Email Phase

The Email Phase ran from March 24, 2022, to April 30, 2022, and entailed testing and full rollout phases. Four different email templates were tested with ∼4600 women Veterans in each group. The most successful template, determined by largest percent of enrollments, was used to contact an additional 478,979 women Veterans. Women were contacted using the same email two times, about 1 week apart.

To determine the impact of the Email Phase, the enrollment rate of the 478,979 women Veterans contacted by email was calculated. Comparisons of the enrollment rate across different demographic groups were conducted using a multivariate adjusted logistic regression model mutually adjusted for age, ethnicity, race, geographic area, and service era. Likelihood of enrollment comparing different groups was calculated using odds ratios (ORs) and 95% confidence intervals (CIs). Missing data were not included in the analyses of enrollment rate but were included in the multivariate adjusted logistic regression model as a separate group.

Data for both phases include information from participant self-report (through the MVP Baseline Survey) supplemented with information obtained from the VA Corporate Data Warehouse (CDW).^9^ All analyses were completed using SAS 9.4 (Cary, North Carolina).

## Results

Overall, 4594 women Veterans enrolled during the MVP Women's Campaign time frame. Of these enrollments, 54% occurred during the Multimedia Phase and 46% occurred during the Email Phase.

### Multimedia Phase

During the 6 months of the Multimedia Phase, a total of 2496 women Veteran enrolled online. The percentage of women enrollees increased from 9.2% to 25.4% of all new enrollees. Table 1 presents the demographic characteristics of women Veterans who enrolled online before (*n*=1068) and after the Multimedia Phase (*n*=2496). Among women Veterans who enrolled online, the percentage of older women enrollees increased, primarily in the 60–69 years age bracket (22.7–35.3%). Women from the southwest (14.5–17.4%, *p*=0.03) and west increased (17.4–23.0%, *p*=0.001) while women from the northeast decreased (12.3–8.4%, *p*=0.01).

**Table 1.** Demographic Characteristics of Women Veteran Online Enrollees Before and After the Multimedia Phase

Characteristics^a^	Total women enrolled online ***n***=3568	Before Multimedia Phase (August 2019–February 2021) ***n***=1072	After Multimedia Phase (March 2021–August 2021) ***n***=2496	Crude ***p***^b^	Multi-***p***^c^
Duration, months		18	6		
Enrollment rate, *n* per month		60	416		
Age at MVP enrollment, mean±SD	52.5±11.4	51.2±11.7	53.0±11.3	<0.001	<0.001
Age category, *n* (%)				<0.001	
18–39 years	597 (16.8)	203 (18.9)	394 (15.9)		Ref
40–49 years	807 (22.7)	272 (25.4)	535 (21.5)		0.42
50–59 years	874 (24.6)	298 (27.8)	576 (23.2)		0.30
60–69 years	1120 (31.5)	243 (22.7)	877 (35.3)		<0.0001
70+ years	160 (4.5)	56 (5.2)	104 (4.2)		0.01
Hispanic, *n* (%)	252 (7.6)	73 (7.2)	179 (7.7)	0.60	0.96
Race, *n* (%)				0.91	
White	2810 (82.4)	850 (81.5)	1960 (82.8)		Ref
Black	400 (11.7)	133 (12.8)	267 (11.3)		0.37
Asian	36 (1.1)	12 (1.2)	24 (1.0)		0.70
Native American/Alaskan	34 (1.0)	9 (0.9)	25 (1.1)		0.70
Native Hawaiian or other Pacific Islander	17 (0.5)	5 (0.5)	12 (0.5)		0.83
Other	27 (0.8)	9 (0.9)	18 (0.8)		0.72
Multiple races	87 (2.6)	25 (2.4)	62 (2.6)		0.61
Geographic area, *n* (%)				<0.0001	
Southeast	1098 (33.8)	371 (35.4)	727 (33.0)		Ref
Northeast	313 (9.6)	129 (12.3)	184 (8.4)		0.01
Midwest	607 (18.7)	209 (19.9)	398 (18.1)		0.91
Southwest	534 (16.4)	152 (14.5)	382 (17.4)		0.03
West	688 (21.2)	182 (17.4)	506 (23.0)		0.001
Territories	10 (0.3)	6 (0.6)	4 (0.2)		0.18
Service era, *n* (%)				0.03	
Sep 2001 or later	240 (7.7)	82 (8.4)	158 (7.4)		0.004
August 1990–August 2001 (including Gulf War)	1211 (38.9)	377 (38.5)	834 (39.1)		Ref
May 1975–July 1990	515 (16.6)	154 (15.7)	361 (16.9)		0.05
August 1964–April 1975 (Vietnam Era)	181 (5.8)	71 (7.2)	110 (5.2)		<0.0001
December 1941–July 1964	11 (0.4)	7 (0.7)	4 (0.2)		0.005
Multiple service eras	954 (30.7)	289 (29.5)	665 (31.2)		0.68

^a^ List of missing observations: age: 10, ethnicity: 229, race: 157, region: 318; service era: 456.

^b^ Comparison between enrolled before and after Multimedia Phase by *t*-test for continuous variable and chi-square test for categorical characteristics.

^c^ Mutually adjusted for age, ethnicity, race, geographic area and service era applying logistic regression model.

MVP, Million Veteran Program; SD, standard deviation.

The percentage of women from the Vietnam era decreased (7.2–5.2%, *p*<0.0001) as well as those who served earlier than July 1964 (0.7–0.2%, *p*=0.005), while women who served during May 1975–July 1990 marginally increased (15.7–16.9%, *p*=0.05). Significant differences for women Veteran online enrollment across different ethnicity and race groups were not observed.

### Email Phase

Of the 478,979 women Veterans contacted as part of the Email Phase, 2098 enrolled between March 2022 and April 2022, representing a 0.44% enrollment rate. Most enrollments occurred online compared to in person (75.3% vs. 24.7%). Table 2 displays the demographic characteristics of women Veterans contacted during the Email Phase. The enrollment rate increased with age increment, especially among the 60–69 years age bracket, who were eight times more likely to enroll into MVP (OR=8.45, 95% CI: 7.31–9.77, *p*<0.0001) compared to women Veterans aged 18–39 years. Compared to White women Veterans, Blacks, Asians, and Native Americans were significantly less likely to enroll, while Veterans with multiple races were more likely to enroll (all *p'*s<0.0001).

**Table 2.** Demographic Characteristics of Women Veterans Contacted During the Email Phase

Characteristics^a^	Veterans with Email delivered^b^	Not enrolled^c^	Enrolled^c^	OR (95% CI)^d^	***p*** ^d^
*n*	478,979	476,881	2098		
%		99.56	0.44		
Age at enrollment, mean±SD	48.1±13.1	48.1±13.1	55.5±10.7		<0.0001
Age category, *n* (%)					
18–39 years	150,176 (31.5)	149,917 (99.8)	259 (0.2)	1.0	Ref
40–49 years	110,825 (23.3)	110,551 (99.7)	274 (0.3)	1.46 (1.23–1.73)	<0.0001
50–59 years	101,488 (21.3)	101,178 (99.7)	310 (0.3)	2.23 (1.88–2.64)	<0.0001
60–69 years	91,467 (19.2)	90,272 (98.7)	1195 (1.3)	8.45 (7.31–9.77)	<0.0001
70+ years	22,555 (4.7)	22,500 (99.8)	55 (0.2)	2.62 (1.92–3.57)	<0.0001
Hispanic, *n* (%)					
No	391,461 (90.5)	389,658 (99.5)	1803 (0.5)	1.0	Ref
Yes	41,317 (9.6)	41,143 (99.6)	174 (0.4)	0.96 (0.81–1.14)	0.65
Race, *n* (%)					
White	258,737 (61.2)	257,272 (99.4)	1465 (0.6)	1.0	Ref
Black	137,736 (32.6)	137,364 (99.7)	372 (0.3)	0.50 (0.44–0.56)	<0.0001
Asian	8838 (2.1)	8810 (99.7)	28 (0.3)	0.59 (0.41–0.86)	0.007
Native American/Alaskan	5192 (1.2)	5176 (99.7)	16 (0.3)	0.57 (0.35–0.94)	0.03
Native Hawaiian or other Pacific Islander	5544 (1.3)	5525 (99.7)	19 (0.3)	0.76 (0.48–1.21)	0.25
Other	427 (0.1)	405 (94.9)	22 (5.2)	1.37 (0.87–2.16)	0.17
Multiple races	6180 (1.5)	6090 (98.5)	90 (1.5)	1.39 (1.11–1.73)	0.004
Geographic area, *n* (%)					
Southeast	138,226 (41.1)	137,522 (99.5)	704 (0.5)	1.0	Ref
Northeast	27,801 (8.3)	27,654 (99.5)	147 (0.5)	0.88 (0.73–1.05)	0.15
Midwest	51,789 (15.4)	51,497 (99.4)	292 (0.6)	0.96 (0.83–1.10)	0.55
Southwest	59,868 (17.8)	59,562 (99.5)	306 (0.5)	0.95 (0.83–1.09)	0.46
West	56,900 (16.9)	56,558 (99.4)	342 (0.6)	1.03 (0.90–1.18)	0.67
Territories	1654 (0.5)	1649 (99.7)	5 (0.3)	0.49 (0.20–1.19)	0.12
Service era, *n* (%)					
September 2001 or later	3201 (1.0)	2960 (92.5)	241 (7.5)	6.30 (5.32–7.47)	<0.0001
August 1990–August 2001 (including Gulf War)	172,721 (51.5)	172,164 (99.7)	557 (0.3)	1.0	Ref
May 1975–July 1990	53,946 (16.1)	53,666 (99.5)	280 (0.5)	0.67 (0.57–0.77)	<0.0001
August 1964–April 1975 (Vietnam Era)	19,185 (5.7)	19,111 (99.6)	74 (0.4)	0.50 (0.38–0.64)	<0.0001
December 1941–July 1964	2098 (0.6)	2094 (99.8)	4 (0.2)	0.36 (0.13–0.97)	0.04
Multiple service eras	84,346 (25.1)	83,708 (99.2)	638 (0.8)	2.66 (2.37–2.99)	<0.0001

^a^ List of missing observations: age: 2468, sex: 2466, ethnicity: 46,201, race: 56,325, region: 142,741; service era: 143,482.

^b^ Percentage is the column percentage of categories proportion.

^c^ Percentage is raw proportion, for example, “Enrolled” and “Not Enrolled” rates within each category of demographic characteristics.

^d^ Mutually adjusted for age, ethnicity, race, geographic area, and service era applying logistic regression model.

CI, confidence interval; OR, odds ratio.

The enrollment rate was significantly higher among women Veterans who served post 2001 (enrollment rate=7.5%, OR=6.30, 95% CI: 5.32–7.47, *p*<0.0001) and those who served in multiple eras (OR=2.66, 95% CI: 2.37–2.99, *p*<0.0001), but significantly lower among women Veterans who served in the Vietnam era (OR=0.50, 95% CI: 0.38–0.64, *p*<0.0001) or between May 1975 and July 1990 (OR=0.67, 95% CI: 0.57–0.77, *p*<0.0001), compared with women Veterans who served in the Gulf War era. We did not observe significant differences of enrollment rates between Hispanic and non-Hispanics, or cross geographic areas (all *p'*s>0.05).

## Discussion

The MVP Women's Campaign represents the first large-scale outreach effort focusing on recruitment of women Veterans into MVP. Through a combination of multimedia outreach and direct email recruitment, MVP was able to significantly increase the number of women Veterans online by over fivefold during a 7-month period.

During the 6 months of the Multimedia Phase, a higher percentage of women Veterans enrolled online who were older (primarily between the ages of 60–69 years) and geographically located in the west. Research from 2017 suggests that Veterans 65 years and older are more likely than their non-Veteran counterparts to go online,^10^ potentially explaining an increase in online enrollment patterns among older women Veterans aged 60–69 years during the Multimedia Phase. Decreases for online enrollment were observed among women Veterans in the northeast, and those who served before 1975. Differences in online enrollment by women Veterans were not observed for ethnicity or race, similar to research indicating that overall, significant differences across ethnicity and race are not reported for internet usage.^11^

Findings from the 1 month of the Email Phase demonstrated increases in White women Veterans and those between the ages of 60–69 years. Decreases were observed for Black women Veterans, with no differences for ethnicity or geographic locale. Despite the higher enrollment observed during the Multimedia Phase compared to the Email Phase, it is important to note the difference in timing between the two phases (6 months vs. 1 month, respectively), suggesting that direct recruitment by MVP through email is a more effective digital outreach mechanism.

The availability of online enrollment offers women Veterans the option to participate at home without having to navigate the multiple challenges reported by women Veterans at VA facilities, such as feeling uncomfortable or experiencing harassment.^4^ With the addition of at-home blood collection capabilities now available for MVP, complete remote enrollment is possible. Given that women Veterans are more likely to endorse use of remote care options,^12^ combined with efforts to better accommodate women Veterans within VA research,^13^ MVP women-focused recruitment efforts will continue to highlight the benefits of online enrollment. In addition, this work supports the development of focused campaigns to reach other populations of interest such as minority Veterans through multimedia and email campaigns tailored to those populations. Doing so can enhance the data available to researchers for addressing health disparities in traditionally underrepresented populations.

Limitations of this work include a comprehensive understanding of how many women Veterans were reached through the Multimedia Phase. Efforts are underway to better monitor and measure the impact of digital MVP outreach and public relations efforts on gender diversification to get a better representation of the denominator. In addition, at the time of the Email Phase deployment, features to assess email open and click rates were unavailable (important tools to understand patterns in email user behavior), thereby limiting the understanding of how many women Veterans acted upon receipt of the emails. As these features have since been made available, future efforts will better describe overall and group specific behavior. General recruitment and enrollment rates for online enrollment (not just online recruitment) are difficult to determine, given the lack of available published information.

## Conclusions

The MVP Women's Campaign demonstrates the ability to utilize tailored digital outreach and engagement methods to successfully connect with specific audiences, ultimately increasing the rate of that audience enrolling in MVP. Attention to messaging and communication channels, combined with a better understanding of effective recruitment methods for certain Veteran populations, allows MVP the opportunity to advance health and health care not only for women Veterans but also beyond. Lessons learned will be applied to increase other populations in MVP such as Blacks, Hispanics, Asians, Native Americans, younger Veterans, and Veterans with health conditions of interest.

## Abbreviations Used

CDW: Corporate Data Warehouse
CI: confidence interval
MVP: Million Veteran Program
OR: odds ratio
SD: standard deviation
VA: Veterans Affairs
VAMCs: VA medical centers
VHA: Veterans Health Administration
VSOs: Veteran-focused Veteran Service Organizations
